# Intra- and Post-Operative Bacteriological Surveys of Surgical Site in Horses: A Single-Centre Study

**DOI:** 10.3390/microorganisms13040928

**Published:** 2025-04-17

**Authors:** Anna Cerullo, Matteo Riccardo Di Nicola, Nicola Scilimati, Alice Bertoletti, Giuseppe Pollicino, Barbara Moroni, Marco Pepe, Sara Nannarone, Rodolfo Gialletti, Fabrizio Passamonti

**Affiliations:** 1 Istituto Zooprofilattico Sperimentale del Piemonte, Liguria e Valle d’Aosta, Via Bologna 148, 10154 Turin, Italy; anna.cerullo@izsplv.it (A.C.); barbara.moroni@izsplv.it (B.M.); 2 Department of Veterinary Science, University of Parma, Strada del Taglio 10, 43126 Parma, Italy; nicola.scilimati@yahoo.it (N.S.); rodolfo.gialletti@unipr.it (R.G.); 3 Department of Veterinary Medicine, Sport Horse Research Centre, University of Perugia, Via San Costanzo 4, 06126 Perugia, Italy; alicebertoletti1@gmail.com (A.B.); marco.pepe@unipg.it (M.P.); sara.nannarone@unipg.it (S.N.); fabrizio.passamonti@unipg.it (F.P.); 4 AniCura Istituto Veterinario Novara, Strada Provinciale 9, Granozzo con Monticello, 28060 Novara, Italy; giuseppepollicinovet@gmail.com

**Keywords:** equine surgery, bacterial contamination, prophylactic measures, surgical site infection, veterinary bacteriology

## Abstract

Bacterial contamination of the surgical site in horses is a major risk factor for the development of surgical site infections (SSIs), which increase morbidity, mortality, the hospitalisation period, antibiotic use, and management costs. While contamination is a prerequisite for infection, its progression to clinical infection depends on additional factors that compromise host defences. The present study, conducted at the Veterinary Teaching Hospital of the University of Perugia over an 11-month period, investigated bacterial contamination in 70 surgeries (53 clean and 17 clean-contaminated) at the end of the procedure. To exclude pre-existing contamination, a sterile swab was collected after surgical scrub, and only cases that entered surgery with a sterile operative field were considered. A swab, biopsy, and fine-needle aspiration from the wound margins were then performed at the end of the surgery to conduct a qualitative assessment of the bacterial contamination of the surgical sites. Risk factors for surgical field contamination were analysed separately for clean and clean-contaminated procedures. Specifically, for clean-contaminated surgeries, the presence of emergency conditions, surgery duration, and intra-operative complications were evaluated. For clean surgeries, risk factors included the type of operating room, surgical duration, tissue involved, use of local anaesthetics, and placement of surgical drapes. The results revealed bacterial contamination rates of 49.1% in clean surgeries and 41.2% in clean-contaminated surgeries. Coagulase-negative staphylococci were the most frequently isolated bacteria, followed by *Burkholderia cepacia*, *Bacillus* sp., and *Stenotrophomonas maltophilia*. A statistical analysis showed no significant results on the predictive factors of the contamination evaluated. However, the observed trends suggest the importance of further investigating these risk factors in a larger sample size. These results emphasise the importance of effective prophylactic measures to limit surgical site contamination. Future research will focus on optimising pre-operative and intra-operative prophylaxis strategies to reduce bacterial contamination to sub-pathogenic levels, thereby enhancing post-operative outcomes.

## 1. Introduction

Historically, surgical site infections (SSIs) were defined as “surgical wound infections”, referring to infections localised to the incision site. In 1992, the Centers for Disease Control and Prevention (CDC) revised this definition to include infections that affect not only the superficial tissue layers but also deeper anatomical structures, adjacent organs, or cavities [1]. Thus, SSIs are defined as infections that occur within 30 days after surgery, or up to one year in patients with implants, at the site of the surgical incision or in the surrounding tissue, organ, or space [1,2,3]. Despite significant advancements in prophylactic measures and surgical techniques, SSIs continue to pose a substantial clinical challenge in both human and veterinary medicine [4,5,6], resulting in among the most common healthcare-associated infections after surgery [3,6,7]. Furthermore, these complications lead to further issues, as they are associated with increased hospitalisation length, healthcare costs, and increased risk of readmission or surgical revision [3,8]. The treatment of SSIs imposes a considerable burden both in human and veterinary medicine, often becoming the critical factor determining the success of a surgical outcome.

Bacterial contamination is the primary risk factor for the development of SSIs. Therefore, the classification of surgical wound types can help predict wound-related complications. The wound classification system, developed as part of the National Research Council’s wound classification criteria, is based on the level of intra-operative contamination and is widely used in both human and veterinary medicine. According to this system, surgical wounds are classified as clean, clean-contaminated, contaminated, or infected [9].

In equine surgery, the incidence of SSIs can range from 0% to 8% in clean orthopaedic procedures, up to 52% in dirty orthopaedic surgeries, and up to 9% in elective laparotomies [10]. Furthermore, in horses undergoing colic surgery, SSIs are a common cause of post-operative morbidity, with an incidence ranging from 3% to 40% [11,12,13,14,15,16,17,18,19,20,21,22,23,24,25,26]. These infections result in prolonged hospitalisation, increased treatment costs, and a higher risk of incisional hernia formation [15,27,28], which is 4–9 times more likely to develop in horses with an incisional infection [27,28]. Thus, infection of laparotomy wounds is a significant issue that could affect the success of the surgical outcomes. Furthermore, in orthopaedic surgeries, the effect of SSIs could be devasting, causing fatal complications such as contralateral-limb laminitis [29] or septic arthritis [30], with financial implications associated with additional specific treatments. Therefore, understanding the ways in which the risk of SSIs can be reduced is of key importance, and the identification of the most important risk factors is essential to prevent the development of these complications. Several risk factors for SSI occurrence have been identified, although the data regarding them are often contradictory because of a lack of standardisation in defining and reporting post-operative complications, particularly in horses undergoing colic surgery [15,26]. The preparation of the patient, surgeon, and surgical environment [12,31], and the presence of concomitant diseases, age, weight [17], duration of colic signs prior to presentation, level of pain at admission [32], intra-operative hypothermia and hypoxia [22], duration and type of surgery [17,32], and suture pattern used for incisional closure [12,18,22] are some of the recognised pre-operative and intra-operative risk factors for SSIs in colic surgeries. The extension of the wound [30] or the reproductive status could be related to a higher risk of SSIs [29] in elective surgeries, while no association was observed with the weight or age class of patients. Furthermore, the use of an abdominal bandage and the type of abdominal bandage used have been identified to influence the development of SSIs [32], and the occurrence of other post-operative complications (e.g., post-operative colic or post-operative reflux) also play crucial roles [15] in the post-operative period of horses who underwent celiotomy.

Bacterial contamination of the surgical site is a necessary condition for infection to occur [6]. However, it is essential to consider a complex interplay of multiple factors, including microbial characteristics (e.g., virulence and pathogen load), host characteristics (e.g., immune status and age), and wound characteristics (e.g., tissue type, extent, and location) [10]. Some previous studies have reported that intra-operative bacterial contamination is not predictive of subsequent SSI [20,33]. Despite this, bacteriological contamination of the surgical wound may represent one of the most significant risk factors, particularly when the bacteria involved exhibit antimicrobial resistance [34], irrespective of whether the contamination occurs during surgery or post-operatively. Since the risk of developing the SSI depends also on the extent of wound contamination with virulent bacteria, efficient prophylaxis is crucial to reduce the number of potential contaminants [35]. Among the most isolated bacteria in SSIs are *Staphylococcus* spp., *Enterococcus* spp., and other bacteria typically belonging to the opportunistic endogenous flora [15]. However, contamination patterns can vary based on several factors, including the surgical and hospital environment, as well as the different prophylactic measures used [31]. The presence of antibiotic-resistant bacteria at the surgical site is relatively uncommon. However, their increasing prevalence, particularly in hospitalised horses, leads to the evaluation of alternative treatment strategies, in addition to the implementation of more effective prophylactic measures [36]. Therefore, reducing bacterial load and early identification of the most common bacteria involved should help reduce the risk of infection and guide appropriate antimicrobial therapy stewardship.

The present study aimed to perform a qualitative bacteriological evaluation of the surgical sites in horses undergoing various types of surgery in an Italian Veterinary Teaching Hospital. The primary aim was to evaluate the bacterial population and to identify potential factors contributing to bacterial contamination of the surgical site in clean and clean-contaminated surgeries in horses, respectively. A secondary aim was to compare the effectiveness of three distinct techniques—cutaneous swabs, fine-needle aspiration, and skin biopsy—in detecting bacterial contamination.

## 2. Materials and Methods

### 2.1. Inclusion Criteria and Data Collection

The study included horses undergoing clean or clean-contaminated surgeries at the Veterinary Teaching Hospital of the University of Perugia over an 11-month period. Informed consent was obtained from all owners. Eligible horses were those undergoing only clean or clean-contaminated procedures, with standardised pre-operative preparation (i.e., shaving of the surgical site, and a preliminary scrub with 4% chlorhexidine digluconate soap before entering the surgical room, followed by a sterile surgical scrub). A sterile swab was obtained from all patients after the surgical scrub, and any patient with bacterial contamination detected in the pre-operative swab was excluded from the study. Horses with a prior surgical history at the same surgical site were also excluded. Data regarding signalment and medical history were recorded. During the intra-operative phase, patients were categorised into two groups based on whether they underwent clean or clean-contaminated surgeries. Due to the differing risk factors associated with each group, variables potentially contributing to surgical site contamination were analysed individually to assess their specific impact.

#### Variables in Clean and Clean-Contaminated Surgeries

In surgeries classified as “clean”, the following factors were considered: the type of tissues involved (hard or soft), the use of local anaesthetics (yes/no), whether the adhesive drape with povidone-iodine used was in place (specifically, it assesses whether the adhesive drape remains attached to the skin along the edges of the surgical wound or not) at the end of the surgery (yes/no), the duration of the procedure, and the surgical room where the surgery was performed (orthopaedic surgery room with ventilation system, non-orthopaedic surgery room with ventilation system, or room without ventilation system).

In “clean-contaminated” surgeries, the following variables were assessed: the presence of emergency conditions (yes/no), occurrence of intra-operative complications such as haemorrhage or hypoxia (yes/no), and duration of the procedure.

### 2.2. Surgical Procedure

**Figure 1:**
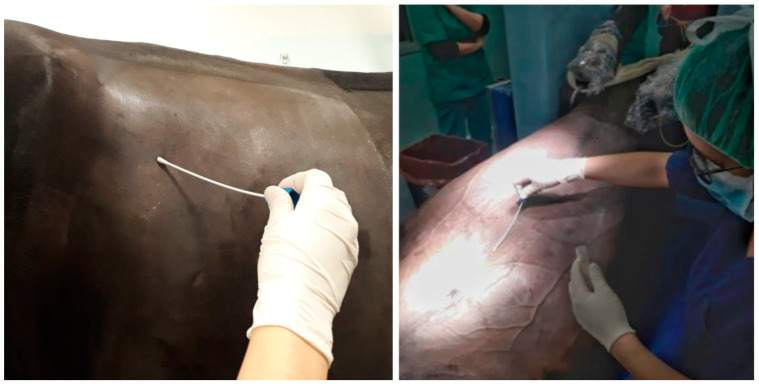
Pre-operative sterile swab collection at the presumed incision site.

Routine patient preparation was carried out, involving a trichotomy of the surgical site using a clipper followed by an initial scrub with 4% digluconate chlorhexidine soap before entering the surgical room. Antimicrobial prophylaxis was performed about 30 min before entering the surgical room. A second surgical scrub was then administered in the surgical room in accordance with good hygiene practices using 4% chlorhexidine soap, and, after 10 min, alcohol-based 0.5% chlorhexidine digluconate. At this point, a sterile skin swab was collected by performing clockwise and counterclockwise circular movements on the presumed incision site for an application time of 5 s (Figure 1).

**Figure 2:**
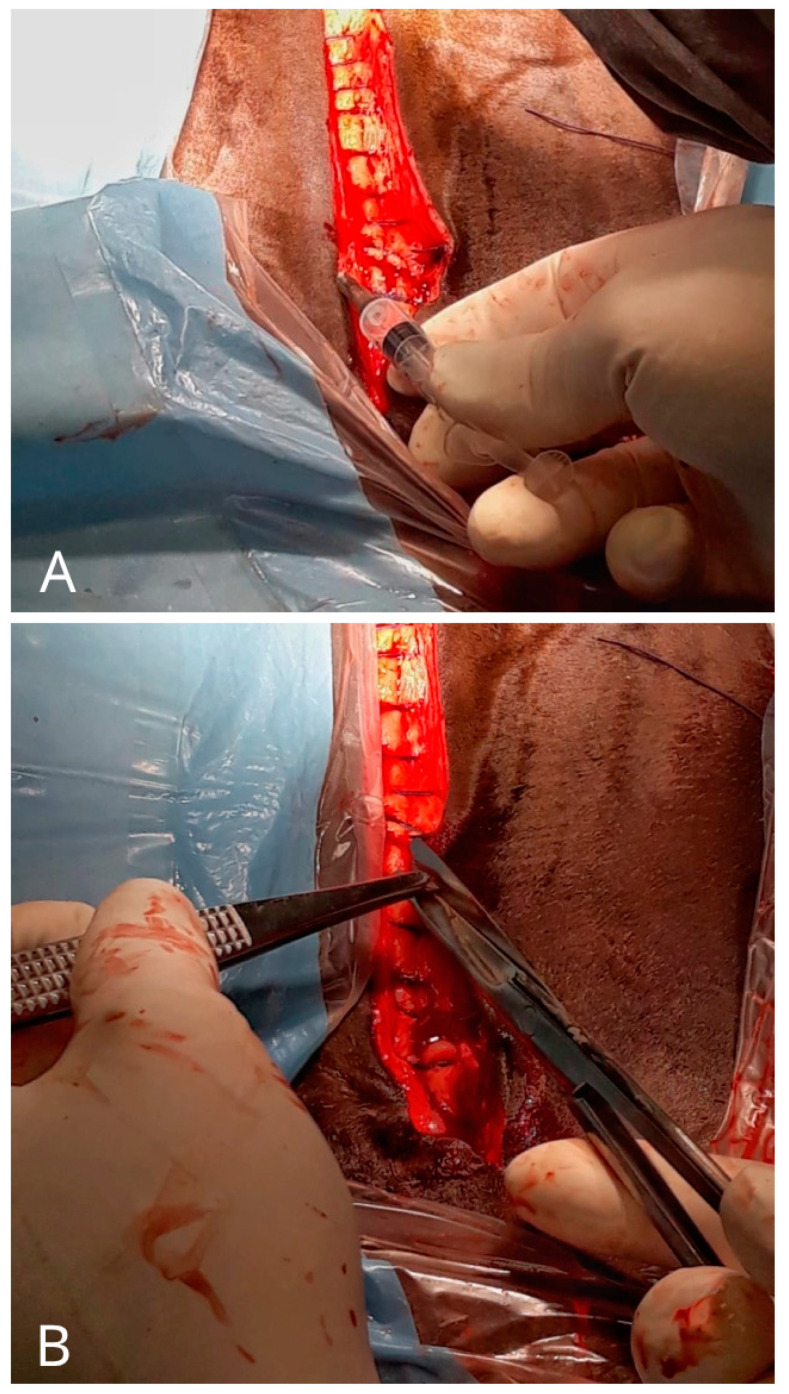
Execution of a fine-needle aspiration (**A**) and biopsy (**B**) on the wound margins.

The surgeons prepared as per routine sterile protocols, ensuring strict asepsis throughout the procedure. A high-cycle autoclave-sterilised surgical set was used, carefully arranged on the instrument table by the surgical assistant. All staff members wore surgical masks, disposable caps, shoe covers, and dedicated surgical scrubs. The surgeons wore a double pair of sterile gloves to minimise the contamination risk. Additionally, sterile drapes were used to isolate the incision site. At the end of the surgery, samples were collected for microbiological assessment using three methods: sterile skin swabs, fine-needle aspiration, and skin biopsies from the wound margins (Figure 2). A sample was classified as “contaminated” if at least one diagnostic method yielded a positive result in the microbiological evaluation.

### 2.3. Bacteriological Evaluation

Swabs collected at the beginning and at the end of surgery, as well as samples obtained from biopsies and fine-needle aspirations, were initially placed in 1 mL of TSB (Tryptone Soya Broth, Thermo Fisher Scientific, Waltham, MA, USA) and incubated at 37 °C for 24 h. Subsequently, they were streaked onto Blood Agar, Mannitol Salt Agar, and MacConkey Agar plates (Neogen Corporation, Lansing, MI, USA). Isolated colonies were assessed based on their morphological, staining, and biochemical characteristics, including Gram staining, catalase, oxidase, and coagulase reactions. Finally, bacterial identification was performed using miniaturised biochemical systems (API^®^ Biomerieux, Marcy-l’Étoile, France) according to the manufacturer’s instructions.

### 2.4. Statistical Analyses

Descriptive statistics were generated for data regarding the age, sex, and weight of the horses admitted, and normality of data was assessed using the Shapiro–Wilk test. Mean (±standard deviation) or median [range] was reported for these data as appropriate. The overall percentage was reported for the type of bacteria isolated in both groups. The differences in results among swabs, biopsies, and fine-needle aspirations during the post-operative phase were also recorded and analysed through a chi-squared test of independence.

Generalised linear models (GLM) with a binomial distribution were employed to evaluate predictive factors influencing wound contamination. Two separate datasets were employed: one focusing on clean surgeries, and the other on clean-contaminated procedures. The logit link function within GLMs to model the probability of contamination events was used, allowing the conversion of the predictors’ linear combination into a log odds format. Initial models included each variable as a linear term to capture their direct effects on the likelihood of surgical contamination. To adequately address the potential non-linear relationships, particularly with continuous variables such as surgery duration, quadratic terms were introduced in subsequent models. Model fit and complexity were assessed using pseudo R-squared values and the Akaike Information Criterion (AIC). Significance was set at *p* < 0.05. The statistical analyses were conducted using the statsmodels package in Python v. 3.13 [37].

## 3. Results

### 3.1. Data Collection and Descriptive Analysis

**Figure 3:**
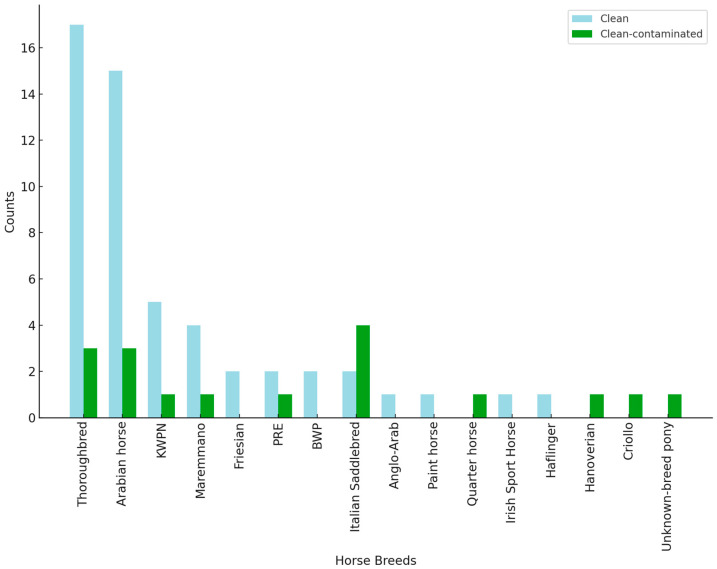
Horse breeds involved in the present study. KWPN = Koninklijk Warmbloed Paard Nederland; PRE = Pura Raza Española; BWP = Belgishe Warmbloed Paard.

Out of 72 horses who underwent different surgeries during the study period, 70 fulfilled the study inclusion criteria, while 2 were excluded due to a positive result on the pre-operative swab. Fifty-three horses out of seventy underwent “clean” surgeries, while seventeen underwent “clean-contaminated” surgeries. Details regarding the type of surgeries and signalment data are reported in Table 1, while data related to the breeds are reported in Figure 3.

### 3.2. Bacteriological Evaluation

**Figure 4:**
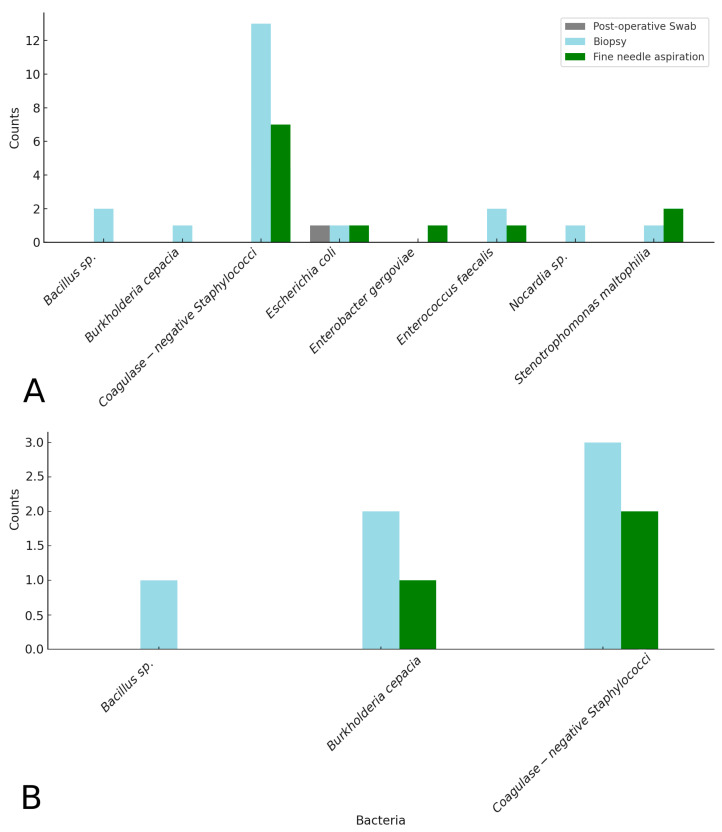
Details on the isolated bacteria by sampling methods in clean (**A**) and clean-contaminated (**B**) surgeries.

Considering the positivity for bacteria in at least one of the three detection methods, 26 out of 53 cases (49.1%) were positive for clean surgeries, and 7 out of 17 cases (41.2%) were positive for clean-contaminated surgeries. Specifically, in clean surgeries, coagulase-negative staphylococci were detected in 16 out of 53 surgeries (30.2%); *Enterococcus faecalis* and *Escherichia coli* were found in 3 cases each (5.7%); *Bacillus* sp. and *Stenotrophomonas maltophilia* were isolated in 2 cases (3.8%); while *Enterobacter gergoviae*, *Nocardia* sp., and *Burkholderia cepacia* were found in 1 case each (1.9%). Details of the isolated bacteria by sampling methods are reported in Figure 4A and Table S1. In clean-contaminated surgeries, coagulase-negative staphylococci were detected in 4 out of 17 surgeries (23.5%), *Burkholderia cepacia* was isolated in 2 cases (11.8%), and *Bacillus* sp. in 1 case (5.9%). Details of the isolated bacteria by sampling methods are reported in Figure 4B and Table S2.

The bacteria detection rates were markedly different across the three methods for all surgical procedures (N = 70): post-operative swabs identified bacteria in only 1 of the 70 surgeries (1.4%), while biopsies and fine-needle aspirations detected bacteria in 27 (38.6%) and 13 cases (18.6%), respectively.

A chi-squared test of independence (df = 2) was conducted to evaluate the statistical significance of the differences observed among the three methods. The chi-squared statistic calculated was 30.792 (*p* = 2.06 × 10^−7^), indicating significant differences in the detection capabilities.

### 3.3. Statistical Analysis

Among the 53 clean surgeries, contamination was detected under different conditions as reported in Table 2. The contaminated surgeries lasted on average 72.2 min (SD ± 44.04), while the non-contaminated surgeries lasted on average 66.3 min (SD ± 36.13).

The analysis of clean and clean-contaminated surgical datasets through GLM with a binomial distribution revealed no statistically significant predictors of surgical contamination, although some trends were observed. Both models exhibited moderate explanatory power, with pseudo R-squared values of 0.164 for clean surgeries and 0.342 for clean-contaminated surgeries, suggesting a modest ability to capture variability in contamination outcomes.

#### 3.3.1. Clean Surgeries

In the clean surgeries’ dataset, the duration of surgery, treated as a continuous variable, displayed a slight negative association with contamination risk (coefficient = −0.0006, *p* = 0.936), although this trend did not reach statistical significance. Local anaesthetics and drape movement also showed non-significant trends, with coefficients of −21.338 (*p* = 0.999) and 1.429 (*p* = 0.162), respectively.

#### 3.3.2. Clean-Contaminated Surgeries

Similarly, the clean-contaminated surgeries’ dataset indicated a slight but not significant positive association between the emergency status and contamination risk (coefficient = 1.641, *p* = 0.259), suggesting that emergency procedures could carry a higher risk. Surgery duration again showed a mild negative, but not significant, trend (coefficient = −0.025, *p* = 0.255). Intra-operative complications, with a coefficient of −0.579 (*p* = 0.703), appeared to have a minimal negative impact on contamination risk.

The lack of significant differences in contamination across various surgical room categories—room for non-orthopaedic surgeries with ventilation system and room without ventilation system compared to room for orthopaedic surgeries with ventilation system—highlighted coefficients of 0.315 (*p* = 0.859) and 21.851 (*p* = 0.999).

## 4. Discussion

Analyses conducted on clean and clean-contaminated surgical datasets using GLM did not identify statistically significant predictors of surgical contamination. However, certain observed trends could impact the prophylactic measures commonly used in equine surgery. In both datasets, the duration of the surgery demonstrated a slightly negative association with the risk of contamination, suggesting that longer surgical procedures do not necessarily entail a higher risk of contamination. This finding is contrary to expectations, as prolonged surgeries typically present a higher infection risk [17,32], which might be presumed to correlate with a greater contamination risk. This can be explained by the fact that longer surgeries are likely those in which greater attention is paid to maintaining asepsis of the surgical site, due to being inherently “riskier”. The onset of SSIs is higher in longer surgeries, probably due to factors other than the degree of contamination at the surgical site, such as an increased risk of trauma to the wound margins [17] or conditions of altered perfusion [22], which are not necessarily related to greater contamination. Moreover, the slight, albeit non-significant, positive association between the emergency status and the risk of contamination in the clean-contaminated surgeries’ dataset raises questions about the intrinsic risks of emergency procedures, as previously noted [17]. In colic surgeries, for instance, the time elapsed for the procedure to begin can significantly impact the outcome [38]. Speed might compromise the accurate application of disinfection protocols, thereby increasing the risk of contaminating the surgical field. However, this does not necessarily affect the development of incisional infections. Furthermore, all horses involved in the study tested negative on the pre-operative swab, thus placing all cases on equal footing in terms of contamination. It is important to note, however, that only the swab was used during the pre-operative phase, which could render the method less sensitive. Similarly, the observed trends regarding surgical drapes, although not statistically significant, suggest that the movement of drapes may be associated with an increased risk of contamination. This implies that procedural modifications could potentially reduce risks. Regarding the use of local anaesthetics, the lack of statistical significance, combined with a *p*-value very close to one, preclude drawing conclusions about the influence of their use on contamination risk based solely on our data.

The findings on environmental contamination, particularly the differences between operating theatres, warrant attention. The theatre without forced ventilation exhibited higher contamination levels compared to those equipped with such systems. Although the data were not statistically significant, this trend supports the notion that air quality in operating theatres can have a substantial impact on the risk of contamination and SSIs, as previously reported in human medicine [39,40]. These results should encourage the standardisation of operating theatres with adequate ventilation systems, especially in veterinary surgery, where standards may not always be consistent.

Another interesting aspect of this study is the identification of the bacterial population from surgical wounds in horses, including coagulase-negative staphylococci, *Burkholderia cepacia*, *Bacillus* sp., and *Stenotrophomonas maltophilia*. The prevalence of coagulase-negative staphylococci is not surprising, as these bacteria are part of the cutaneous microbiota and can persist even after thorough surgical preparation. A previous study in a large sample of horses confirmed the prevalence of *Bacillus* sp. and staphylococci on laparotomy wounds post-surgery [15]. Although their presence does not always indicate pathology, they may pose a risk to animals that have undergone surgery or to immunocompromised animals. This finding reinforces the notion that even bacteria considered “minor” or environmental contaminants can become pathogens under certain conditions, such as during the post-operative period. The detection of *B. cepacia* is particularly significant, given the well-documented resistance of this bacterium to multiple antibiotics and its association with nosocomial infections in both humans [41,42] and veterinary medicine [43,44,45]. *Burkholderia cepacia* is an aerobic, motile, Gram-negative bacterium found ubiquitously in the environment. It can be isolated from soil, water, and plants [46] and is a rapidly growing bacterium that survives on minimal nutritional resources in hostile environments and is resistant to disinfectants [42]. Although it appears to act as a contaminant in this context, its presence raises concerns regarding the management of post-operative infections. Should contamination with *B. cepacia* progress to infection, treatment options would be limited, increasing the risk of complications, costs, and prolonging hospital stays. Another important aspect to consider is that wound swabbing showed extremely low sensitivity compared to biopsy and needle aspiration, yielding a positive result in only one case out of thirty-three total positives. This does not necessarily entail significant implications for the detection of contamination, but it may be important to consider in cases where a difficult-to-treat SSI develops. In such situations, swabbing alone may not be sufficient to isolate the bacteria involved, making it advisable to also consider these other two more invasive diagnostic techniques. Finally, the main result of this study is the high percentage of contamination observed at the end of surgical procedures. This outcome highlights that, despite some surgeries being classified as “clean”, there is a considerable risk of contamination that might be underestimated in clinical protocols. This underscores the importance of adhering to stringent sterilisation practices even in “low-risk” surgeries, as contamination, although not always leading to infections, represents a significant risk factor.

Limitations of the study include the non-detection of parameters that could influence contamination, such as temperature and the seasonality of surgeries. Indeed, temperature and seasonal variations play a significant role in bacterial growth, as environmental conditions—such as humidity and the prevalence of specific pathogens—fluctuate throughout the year. Seasonal changes, particularly during warmer months, often lead to increased fly populations, which may elevate the risk of wound contamination in equine surgeries. This is especially relevant considering that operating rooms are often not equipped with advanced ventilation systems. Future studies should incorporate these variables to assess their impact on surgical outcomes. Additionally, it would be valuable for subsequent research to focus on the molecular characterisation of bacteria isolated during surgical procedures, also evaluating potential pathogenic factors or patterns of antibiotic resistance. In this regard, a longitudinal study monitoring the evolution of contamination during follow-up could help determine whether bacteria isolated at earlier stages are implicated in the development of SSIs.

## 5. Conclusions

The present study, while limited in case number, underscores the importance of examining a wider array of factors across a larger sample to secure more robust findings. Acknowledging the absence of statistically significant results, the observed trends provide critical insights for future research and clinical practices. Both clean and clean-contaminated surgery groups demonstrated similar rates of positive microbiological findings, suggesting that the contamination risks are comparable across different surgical categories. This highlights an opportunity to reconsider the necessity and extent of antibiotic prophylaxis, potentially reducing the reliance on antimicrobials and thereby limiting the risk of antibiotic resistance —an escalating concern in hospital settings. Moreover, the implementation of more precise contamination detection techniques and the standardisation of intra-operative protocols could significantly reduce post-operative infection risks and enhance clinical outcomes for equine patients. This study revealed that despite rigorous pre-operative preparations, a significant rate of bacterial contamination persists, and in addition to advocating for improvements in surgical protocols and environmental controls, it is likely that optimising operating room ventilation could further mitigate infection risks. Overall, future research should focus on expanding the sample size and the post-operative sampling time to better evaluate the risk factors associated with post-operative bacterial contamination. In addition to widening the scope of studies, efforts should be directed toward improving surgical protocols and environmental controls, such as optimising operating room ventilation. Investigating the impact of these factors on infection rates could help refine preventive measures. Long-term studies tracking the development of infections and identifying factors like immune status or pre-existing conditions would also provide valuable insights, ultimately contributing to more effective strategies for minimising bacterial contamination and improving outcomes in equine surgery.

## Figures and Tables

**Table 1:** Data regarding type of surgeries, sex, age, and weight, in “clean” and “clean-contaminated” groups.

Scheme		Sex (n)			Mean Age (±SD)	Median Weight kg [Range]
**Clean (53)**	Laparoscopy (20)	Intact male (25)	Female (23)	Gelding (5)	6.35 (SD ± 6.8)	395 [75–650]
	Arthroscopy (17)					
	Laparotomy without enterotomy/enterectomy (6)					
	Arthrodesis (5)					
	Herniorrhaphy (3)					
	Neurectomy (1)					
	Tie-forward (1)					
**Clean-contaminated (17)**	Laparotomy with enterotomy/enterectomy (17)	Intact male (0)	Female (12)	Gelding (5)	9.68 (SD ± 6.4)	500 [300–650]

**Table 2:** Number of contaminated cases among the various categories considered.

Category		Contaminated Cases
**Type of tissue**	Soft	17 out of 33
	Hard	9 out of 20
**Use of local anaesthetics**	Yes	12 out of 20
	No	14 out of 33
**Drape movement**	Yes	16 out of 27
	No	10 out of 26
**Type of surgery room**	Without ventilation	13 out of 21
	Non-orthopaedic, with ventilation	3 out of 10
	Orthopaedic, with ventilation	10 out of 22

## Data Availability

All data generated or analyzed during this study are included in this published article and its Supplementary Materials.

## Supplementary Materials

The following supporting information can be downloaded at: https://www.mdpi.com/article/10.3390/microorganisms13040928/s1, Table S1: Bacterial detection in clean surgeries (N = 53); Table S2: Bacterial detection in clean-contaminated surgeries (N = 17).
