# Supplementary material for: Intra- and Post-Operative Bacteriological Surveys of Surgical Site in Horses: A Single-Centre Study
Source: Microorganisms. 2025 Apr 17;13(4):928. doi: 10.3390/microorganisms13040928 (PMC12029434; doi:10.3390/microorganisms13040928)
Supplement: Supplementary file 1 [file microorganisms-13-00928-s001.zip › microorganisms-3490608-supplementary.pdf]

Intra- and post-operative bacteriological surveys of surgical site in horses: a single-center study

Anna Cerullo, Matteo Riccardo Di Nicola, Nicola Scilimati, Alice Bertoletti, Giuseppe Pollicino, Barbara Moroni, Marco Pepe, Sara Nannarone, Rodolfo Gialletti and Fabrizio Passamonti

SUPPLEMENTARY FILE

Table S1. Bacterial detection in clean surgeries (N = 53).

Surgery ID	Post-operative swab	Biopsy	Fine needle aspiration
1			
2			
3		Coag. neg. staphylococci	*Enterobacter gergoviae*; Coag. neg. Staphylococci
4		*Stenotrophomonas maltophilia*	*Stenotrophomonas maltophilia*
5		*Escherichia coli*	Coag. neg. staphylococci
6			
7		Coag. neg. staphylococci	
8			
9			
10		*Enterococcus facalis*	
11			
12			
13			
14			*Stenotrophomonas maltophilia*
15		Coag. neg. staphylococci	
16			
17			
18			
19		*Bacillus* sp.	
20			
21			Coag. neg. staphylococci
22			Coag. neg. staphylococci
23		Coag. neg. staphylococci	
24		*Nocardia* sp.	
25			
26	*Escherichia coli*		
27			
28		Coag. neg. staphylococci	Coag. neg. staphylococci
29		*Burkholderia cepacia*	
30		Coag. neg. staphylococci	
31			
32		Coag. neg. staphylococci	
33			*Escherichia coli*
34		*Bacillus* sp.	
35			
36			
37			
38			
39		*Enterococcus faecalis*	
40			
41			
42		Coag. neg. staphylococci	Coag. neg. staphylococci
43		Coag. neg. staphylococci	

44			
45		Coag. neg. staphylococci	*Enterococcus faecalis*; Coag. neg. Staphylococci
46		Coag. neg. staphylococci	
47		Coag. neg. staphylococci	
48			
49			
50			
51		Coag. neg. staphylococci	
52			
53			

Table S2. Bacterial detection in clean-contaminated surgeries (N = 17).

**Surgery ID**	**Post-operative swab**	**Biopsy**	**Fine needle aspiration**
54			
55			
56			
57			
58		*Burkholderia cepacia*	
59			
60			
61		*Burkholderia cepacia*	*Burkholderia cepacia*
62		*Bacillus* sp.	
63			
64			
65			Coag. neg. staphylococci
66		Coag. neg. staphylococci	
67		Coag. neg. staphylococci	Coag. neg. staphylococci
68		Coag. neg. staphylococci	
69			
70			
